# Prognostic value of patient-reported outcome measures (PROMs) in adults with non-small cell Lung Cancer: a scoping review

**DOI:** 10.1186/s12885-022-10151-z

**Published:** 2022-10-19

**Authors:** Kuan Liao, Tianxiao Wang, Jake Coomber-Moore, David C Wong, Fabio Gomes, Corinne Faivre-Finn, Matthew Sperrin, Janelle Yorke, Sabine N van der Veer

**Affiliations:** 1 Centre for Health Informatics, Division of Informatics, Imaging and Data Sciences, Faculty of Biology, Medicine and Health, Manchester Academic Health Science Centre, The University of Manchester, Manchester, UK; 2 Patient-Centred Research Centre, The Christie NHS Foundation Trust, Manchester, UK; 3 Department of Computer Science, University of Manchester, Manchester, UK; 4 Medical Oncology Department, The Christie NHS Foundation Trust, Manchester, UK; 5 The Christie NHS foundation Trust, Manchester, UK; 6 Division of Cancer Science, The University of Manchester, Manchester, UK; 7 Division of Nursing, Midwifery and Social Work, University of Manchester, Manchester, UK

**Keywords:** Clinical decision rules, Lung neoplasms, Non-small cell lung cancer, Patient-reported outcome measures, Prognosis, Review

## Abstract

**Background:**

There is growing interest in the collection and use of patient-reported outcome measures (PROMs) to support clinical decision making in patients with non-small cell lung cancer (NSCLC). However, an overview of research into the prognostic value of PROMs is currently lacking.

**Aim:**

To explore to what extent, how, and how robustly the value of PROMs for prognostic prediction has been investigated in adults diagnosed with NSCLC.

**Methods:**

We systematically searched Medline, Embase, CINAHL Plus and Scopus for English-language articles published from 2011 to 2021 that report prognostic factor study, prognostic model development or validation study. Example data charting forms from the Cochrane Prognosis Methods Group guided our data charting on study characteristics, PROMs as predictors, predicted outcomes, and statistical methods. Two reviewers independently charted the data and critically appraised studies using the QUality In Prognosis Studies (QUIPS) tool for prognostic factor studies, and the risk of bias assessment section of the Prediction model Risk Of Bias ASsessment Tool (PROBAST) for prognostic model studies.

**Results:**

Our search yielded 2,769 unique titles of which we included 31 studies, reporting the results of 33 unique analyses and models. Out of the 17 PROMs used for prediction, the EORTC QLQ-C30 was most frequently used (16/33); 12/33 analyses used PROM subdomain scores instead of the overall scores. PROMs data was mostly collected at baseline (24/33) and predominantly used to predict survival (32/33) but seldom other clinical outcomes (1/33). Almost all prognostic factor studies (26/27) had moderate to high risk of bias and all four prognostic model development studies had high risk of bias.

**Conclusion:**

There is an emerging body of research into the value of PROMs as a prognostic factor for survival in people with NSCLC but the methodological quality of this research is poor with significant bias. This warrants more robust studies into the prognostic value of PROMs, in particular for predicting outcomes other than survival. This will enable further development of PROM-based prediction models to support clinical decision making in NSCLC.

**Supplementary Information:**

The online version contains supplementary material available at 10.1186/s12885-022-10151-z.

## Background

Lung cancer is the second most common cancer, with an estimated 2.2 million patients newly diagnosed worldwide, accounting for 11.4% of all new cancer cases [1]. Non-small cell lung cancer (NSCLC), accounts for approximately 85% of lung cancer cases [2]. NSCLC may cause a range of symptoms (e.g., chronic cough, pain, dyspnoea, fatigue), psychological issues and decreased physical function [3]. This can negatively affects health-related quality of life (HRQOL)[4]. Another reason for this decreased HRQOL is related to anticancer treatments and their associated side effects [4].

Patient-reported outcomes measures (PROMs) are tools to assess patients’ views on aspects of their health and condition, including HRQOL, symptom status, physical function and mental health [5]. In the field of lung cancer, they can be categorised into generic, cancer-specific and lung cancer-specific instruments [6]. They can be used as part of the clinical management of lung cancer, with the aim to improve patient-clinician communication, decision making and patient satisfaction [5]. The increasing use of PROMs as part of clinical care contributes to the paradigm shift from illness-focused to patient-centred care [7]. Randomised controlled trials comparing PROM-directed follow-up to usual care demonstrated that integrating PROMs in care pathways was associated with better symptom control, reduced emergency department attendance and hospitalisation, and improved survival [8, 9].

Decisions regarding the treatment of NSCLC often involve a trade-off between potential benefits (e.g., prolonged survival) and potential risks (e.g., treatment toxicity, decreased HRQOL) [10]. PROMs could inform discussions about this trade-off and support shared treatment decision-making by patients and healthcare professionals [6]. Integrated as predictors into prognostic models, PROMs could also provide insights into patient’s future course of disease based on a current assessment of their own health and condition [11, 12]. However, several steps are required before such prognostic models can be implemented in practice. These include identification of the association between candidate prognostic factors and outcomes, development and validation of the model, and evaluation of its clinical utility and impact [13, 14].

A 2021 systematic review, in line with previous studies [6, 15, 16], suggested PROMs provided prognostic information for overall survival in a range of cancer populations, including lung cancers [17]. However, it is unclear if high quality studies are available that investigated the value of PROMs for predicting all clinical outcomes. This review aimed to address this unmet need by examining to what extent, how and how robustly research has been conducted on the prognostic value of PROMs in patients with NSCLC. As this topic concerns how research has been conducted, one suitable approach, which we adopt, is a scoping review [18]. This review will contribute to understanding and unlocking the potential of PROMs to support and enhance clinical decision making and, ultimately, to improve the outcomes of patients with lung cancer.

## Methods

We conducted a scoping review [18] to identify and characterise published studies that evaluated the association between PROMs (as candidate prognostic factors) and outcomes, or that developed prognostic models including PROMs as predictors. We designed, conducted the review in accordance with the JBI’s guidance for conducting systematic scoping reviews and reported the review in line with the Preferred Reporting Items for Systematic reviews and Meta-Analyses extension for Scoping Reviews (PRISMA-ScR) [19, 20].

### Database search strategy

We systematically searched Medline and Embase via Ovid, CINAHL Plus and Scopus on Aug 9th, 2021. Based on terms used by other published reviews on related topics [21–23], our search syntax combined keywords and MeSH terms related to ‘NSCLC’, ‘PROMs’ and ‘prediction or prognosis’ (see full search syntax in Appendix 1). To complement our electronic search, we manually searched the reference lists of all included studies and relevant reviews.

### Inclusion criteria for relevant studies

The inclusion criteria for assessing the relevance of studies were structured according to the Population, Concept and Context framework for scoping reviews [19]. We deemed articles relevant if (1) the proportion of adults ≥ 18 years old with NSCLC in the study population was 50% or higher, (2) any generic, cancer-specific or lung cancer-specific PROMs or their components were independent variables or predictors, (3) the study entailed prognostic prediction of any future outcome at the individual level, (4) it was a prognostic factor study or prediction model development or validation study, (5) it was a published original study in English with full-text available, including full-text conference papers and (6) published after 2011, as survival of patients with NSCLC improved since owing to recent advances in treatment [24–26]. Appendix 2 presents a detailed overview of inclusion and exclusion criteria.

### Screening and study selection

After removing duplicates, all titles and abstracts were screened independently by two reviewers (KL, TW, JC), with KL screening all and TW and JC each screening half. For potentially relevant studies identified by screening titles and abstracts, two reviewers (KL and TW) assessed the full text independently and in duplicate. Discrepancies were resolved through consensus and discussed with a third reviewer (SNVDV), if needed. Reasons for exclusion were recorded for the full-text screening stage only. Both titles and abstracts and full text screening were conducted using Rayyan (https://www.rayyan.ai/).

### Extracting and charting the results

For data charting, we were interested in what and how PROMs had been collected, what outcomes were evaluated in association with PROMs or predicted by PROMs, and what statistical methods had been used. For this, we used the items suggested by the Cochrane Prognosis Methods Group (https://methods.cochrane.org/prognosis/tools) and a similar prognostic scoping review in another clinical area [27]. We organised, categorised and charted data as follows: characteristics of the study, characteristics of PROMs as predictors, characteristics of outcomes, and statistical methods. Specific data items and their definition are listed in Appendix 3. Two reviewers (KL and SNVDV) tested the data charting form by independently charting the data from a randomly selected eligible study. Two reviewers (KL and TW) charted data independently and in duplicate. They resolved discrepancies in the charted data through discussion and discussed with a third reviewer (MS) if needed. All the data were charted in Excel spreadsheets.

### Collating, summarising, and reporting the results

The extracted evidence was repeatedly reviewed in the process of collating and summarising the findings. Results were synthesised through a descriptive numerical summary analysis to present an overview of the current evidence on the value of PROMs used as prognostic factors.

### Risk of bias assessment

To provide an overview of the quality of available research on the prognostic value of PROMs, we critically appraised included studies using one of two well-established risk of bias tools. For prognostic factor studies, we used the QUality In Prognosis Studies (QUIPS) tool recommended by the Cochrane Prognosis Methods Group [28] and rated the overall risk of bias in each study following the procedure suggested by Grooten and colleagues [29]. For prognostic model development studies, we applied the risk of bias assessment section of the Prediction model Risk Of Bias ASsessment Tool (PROBAST) [30]. KL appraised all studies, with TW appraising a randomly selected 25% in duplicate. Inter-rater agreement was calculated as the percentage of the agreed opinion on the domain level. The inter-rater agreement was 70% and achieved full inter-rater agreement after discussion of discrepancies with the third reviewer. We used the robvis web tool (https://mcguinlu.shinyapps.io/robvis/) [31] to plot risk of bias summary graphs.

## Results

**Fig. 1:**
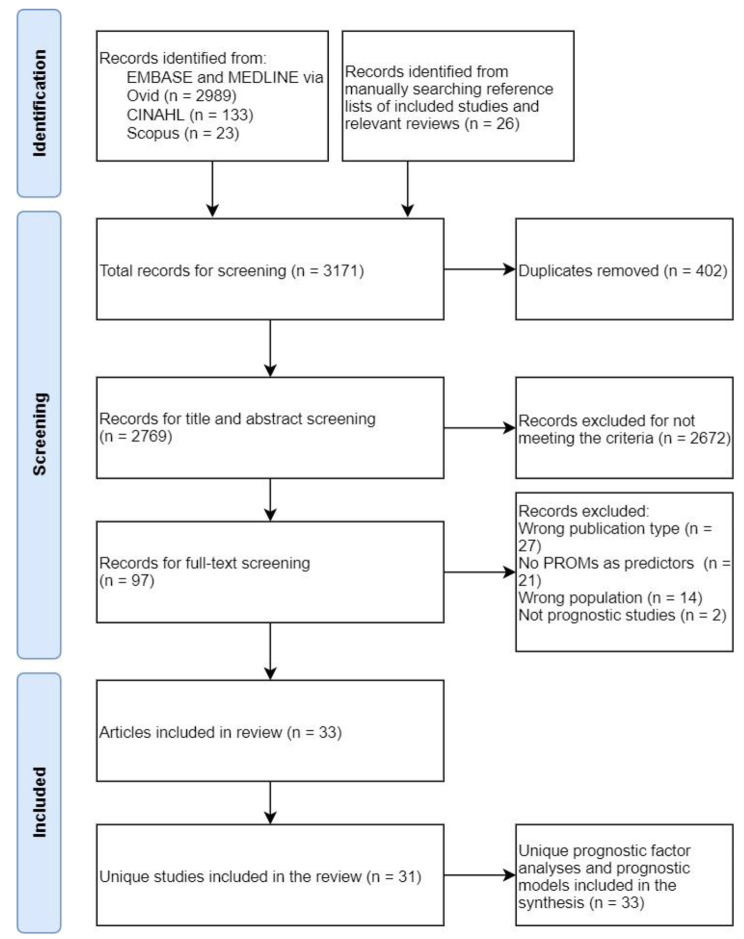
Identification and selection of articles, studies, and prognostic factor analyses and models

Figure 1 shows that our search yielded 2,769 unique titles, of which 97 articles were eligible for full-text screening. The most common reasons for excluded full-text articles were the wrong publication type (n = 27) and the lack of PROMs as predictors (n = 21). Finally, we included 33 articles, reporting the results of 31 unique studies and 33 unique prognostic factor analyses and prediction models (referred to in the remainder of the manuscript as ‘analyses and models’).

### Characteristics of included studies

Table 1 shows the characteristics of the 31 included studies [15, 16, 32–60]. The majority of studies were prognostic factor studies (n = 27, 88%), had a study population consisting of NSCLC patients only (n = 23, 74%), and used data from an observational study (n = 18, 58%). The median sample size was 237.5 (range, 35 to 6290). There were no studies externally validating a prediction model. Study-level information on characteristics is available in Appendix 4.

**Table 1:** The characteristics of included studies (n = 31)

Characteristics	Number of studies (%)
**Type of population**	
NSCLC	23 (74)
Mixed population ^a)^	7 (23)
Not reported	1 (3)
**Sample size**	
< 200	12 (39)
200–500	10 (32)
> 500	9 (29)
**Study design**	
Prognostic factor study	27 (88)
Prognostic model development	4 (12)
**Data source**	
Observational study	18 (58)
Clinical trial	8 (26)
Routinely collected data	5 (16)

Abbreviations: NSCLC, non-small cell lung cancer

a) Proportion of patients with NSCLC in sample > 50%.

### Characteristics of PROMs as predictors in analyses and models

**Fig. 2:**
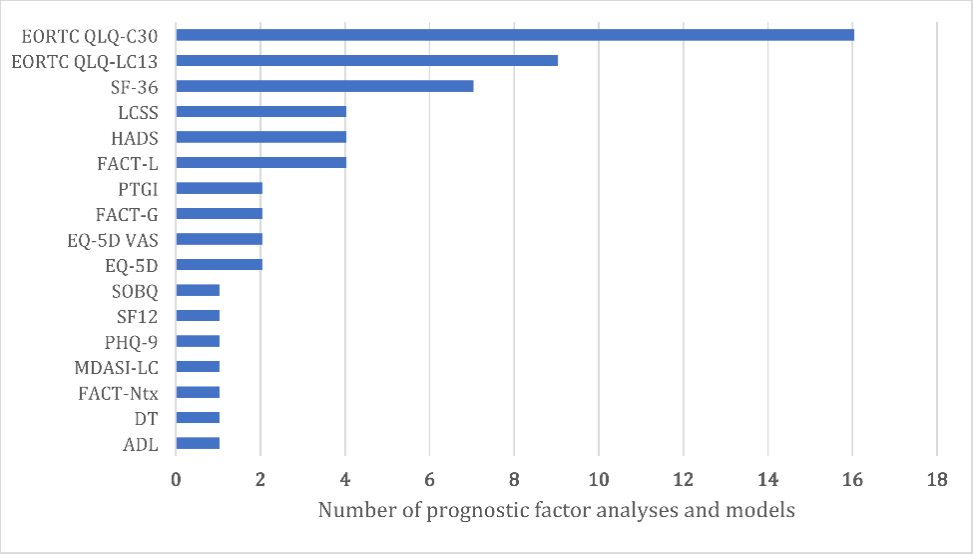
PROMs used as predictors in prognostic factor analyses and prognostic models (n = 33). More than one PROMs could be used in an analysis or model. EORTC QLQ-C30, The European Organization for Research and Treatment of Cancer Quality of Life Questionnaire – Core 30; EORTC QLQ-LC13, EORTC Quality of Life Questionnaire–Lung Cancer Module; SF-36, 36-item Short Form Health Survey; FACT-L, Functional Assessment of Cancer Therapy–Lung; HADS, Hospital Anxiety and Depression Scale; LCSS, Lung Cancer Symptom ScaleEQ-5D, EuroQol-5D; EQ-VAS, EuroQol-Visual Analogue Scale; FACT-G, Functional Assessment of Cancer Therapy–General; PTGI, Posttraumatic Growth Inventory; ADL, Katz’s Activities of Daily Living, DT, Distress Thermometer; FACT-Ntx, Functional Assessment of Cancer Therapy/Gynecologic Oncology Group–Neurotoxicity Questionnaire; MDASI-LC, M. D. Anderson Symptom Inventory - Lung Cancer; PHQ-9, Patient Health Questionnaire-9; SF-12, 12-item Short Form Health Survey; SOBQ, The University of California San Diego Shortness of Breath Questionnaire.

Figure 2 shows the 33 analyses and models (from 31 studies) used a total of 17 different PROMs [15, 16, 32–60]. The European Organization for Research and Treatment of Cancer Quality of Life Questionnaire (EORTC QLQ)– Core 30 (EORTC QLQ-C30) was most commonly used (n = 16, 48%), followed by the EORTC QLQ-Lung cancer module (EORTC QLQ-LC13) (n = 9, 27%) and the 36-item Short Form Health Survey SF-36 (n = 7, 21%).

Table 2 summarises the characteristics of PROMs that were used. Most analyses and models included generic PROMs as predictors (n = 18, 55%), followed by the lung-cancer specific PROMs (n = 16, 48%). HRQOL was the most investigated aspect, measured in nearly all included analyses and models (n = 31, 94%). The majority of the analyses and models applied the subdomain scores (n = 18, 54%) of PROMs and collected PROMs only once at baseline (i.e. preoperative, pretreatment, or at enrolment; n = 24, 73%). Information at the analysis/model level is available in Appendix 5.

**Table 2:** Characteristics of PROMs as predictors in prognostic factor analyses and prediction models (n = 33)^a)^

Characteristics	Number of analyses/ models (%)
**PROM classification**	
Generic	11 (33)
Cancer-specific + lung cancer-specific	7 (21)
Cancer-specific	4 (12)
Lung cancer-specific	4 (12)
Generic + lung cancer-specific	3 (9)
Generic + cancer-specific	2 (6)
Combination of all three classifications	2 (6)
**Construct measured by PROM**	
HRQOL	19 (58)
HRQOL + symptom burden	10 (30)
HRQOL + functional status	2 (6)
Symptom burden	2 (6)
**Type of PROM score**	
Subdomain score	12 (36)
Overall summary score	8 (24)
Single item score	7 (21)
Subdomain score + overall summary score	5 (15)
Single item score + subdomain score	1 (3)
**Cross-sectional vs. change in PROM score**	
Cross-sectional PROMs score	25 (76)
Change in PROMs scores	4 (12)
Both	4 (12)
**Time point of PROM collection**	
Baseline	24 (73)
Baseline + at least once after treatment	6 (18)
After first treatment	2 (6)
Not reported	1 (3)

Abbreviations: PROM, patient-reported outcome measure

a) Möller et al. (2012) and Fernando et al. (2015) each presented two models, which are reported separately in this table.

### Characteristics of predicted outcomes

Almost all analyses and models (n = 32, 97%) aimed to predict overall survival. Only one study focused on predicting self-rated health status (as measured by the EuroQol-Visual Analogue Scale and the EORTC global health status), which were measured 1-year postoperatively [47]. The median follow-up time for these studies ranged from 180.3 days to 4.4 years, and the maximum length of observation ranged from 381 days to 12 years. One model had progression-free survival and treatment-related adverse events as secondary outcomes [44]. Appendix 6 contains analysis/model-level information on the characteristics of predicted outcomes.

### Statistical methods using for prognostic modelling using PROMs

**Fig. 3:**
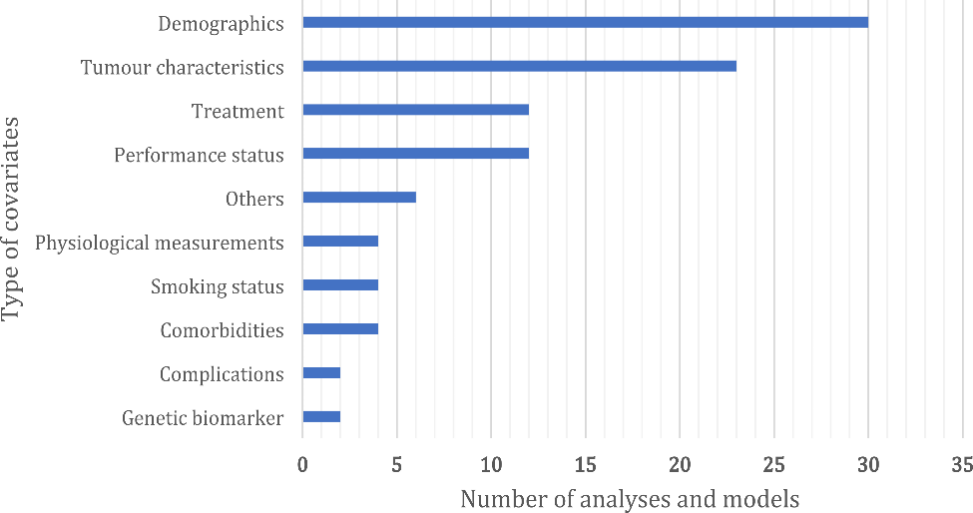
Types of covariates in the prognostic factor analyses and prognostic models (each could include more than one type of covariates); covariates in ‘others’ included, e.g., survey mode of administration (e.g., paper or telephone), C-reactive protein, and forced expiratory volume

Table 3 summarises the characteristics of statistical methods used for analyses and models. Almost all analyses and models (n = 31, 93.9%) fitted a multivariable model. The Cox proportional hazards regression was the most used predictive modelling technique (n = 31, 93.9%). Over half of the analyses and models did not report the selection procedure of predictors in multivariable models (n = 17, 52%). Information on the analysis/model-level is presented in Appendix 7. Figure 3 shows the types of covariates that were included in analyses and models in addition to PROMs, with demographics (n = 30, 91%) and tumour characteristics (n = 23, 70%) being most common. Other types of covariates that were adjusted for in at least in two analyses/models were comorbidities, smoking status, physiological measurements, genetic biomarkers, and complications. Detailed information of covariates on the level of analysis and models can be found in Appendix 7.

**Table 3:** Statistical methods for prognostic factor analyses and prediction models using PROMs (n = 33)a)

Characteristics	Number of analyses/models(%)
**Uni-/multivariable**	
Multivariable	31 (94)
Univariable	2 (6)
**Types of predictive modelling techniques**	
Cox proportional hazards regression	31 (94)
Random forest	1 (3)
Linear regression	1 (3)
**Selection of predictors in multivariable models**	
Not reported or not appliable	17 (52)
Univariable screening	8 (24)
Univariable screening + Automatic selection procedures	4 (12)
Fitting a full model suggested by previous literatures or experts	4 (12)

a) Möller et al. (2012) and Fernando et al. (2015) each presented two prognostic factor analyses, which are reported separately in this table

### Risk of bias assessment

**Fig. 4:**
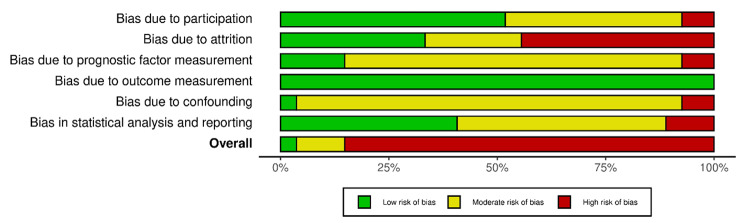
Summary of risk of bias in prognostic factor studies (n = 27). Plotted by the robvis web tool (https://mcguinlu.shinyapps.io/robvis/)

Figure 4 summarises the risk of bias in the included prognostic factor studies (n = 27). Overall, only 1 (4%) had a low risk of bias, whereas 23 (85%) had a high risk of bias, mainly due to bias of handling the existing prognostic factors, of which almost all the studies (n = 26, 96%) had moderate to high bias, except for the study by Greer and colleagues [42]. The vast majority had moderate to high risk of biases due to prognostic factor measurement (n = 23, 85%) and attrition (n = 18, 67%). The results of the risk of bias appraisal at the prognostic factor study level are presented in Appendix 8.

**Fig. 5:**
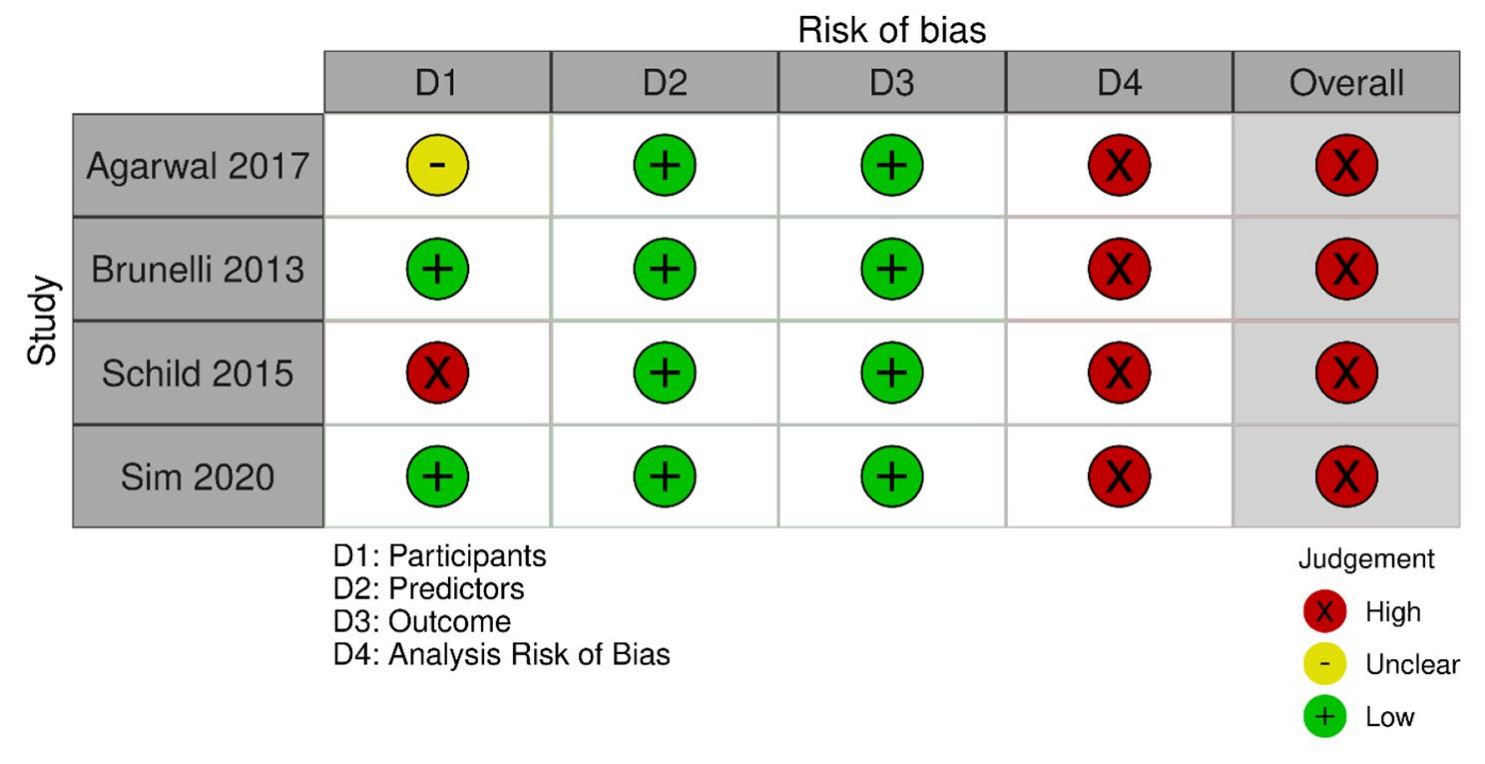
Summary of risk of bias in prediction model development studies (n = 4). Plotted by the robvis web tool (https://mcguinlu.shinyapps.io/robvis/)

The risk of bias in the prognostic model development studies (n = 4) is presented in Fig. 5. The overall risk of bias in all four was high due to the high risk of bias in the statistical analysis, which was usually a result of not reporting the overfitting and optimism in model performance.

## Discussion

This scoping review found that PROMs for prognostic prediction in adults with NSCLC were investigated in a substantial number of studies. The majority were prognostic factor studies, while a few developed prognostic models; no external validation study was identified. Cancer-specific PROMs, such as the EORTC QLQ-C30, and those measured at baseline and summarised as a subdomain score were most frequently used as predictors. Almost all studies used multivariable Cox proportional hazards regression to predict overall survival only, whereas prediction of other clinical outcomes appeared underinvestigated. The risk of bias in included studies was mostly moderate to high.

We found that the EORTC QLQ-C30 was the most frequently used PROMs for prognostic predicting in patients with NSCLC. This finding was consistent with previous reviews [17, 61–63]. This may be explained by the fact that the EORTC QLQ-C30 is also the most commonly used PROM as part of the clinical management of lung cancer [6], meaning that it is a well-established PROM for which routine collection is more likely to be available.

With only one high-quality prognostic factor study identified in this review [36], more high-quality research is warranted to examine the prognostic value of PROMs. Future prognostic studies need to report how the prognostic factors are selected. In the event that factors are selected automatically (data-driven), the method for factor selection should be reported, ideally avoiding univariable selection methods. [64, 65]. Additionally, further studies need to take into account the risks associated with the categorising or dichotomising continuous predictors, which leads to loss of information and increases the risk of false positives [66].

The four prognostic models using PROMs as predictors in our review were all only internally validated [32, 37, 56, 57]. This finding is consistent with previous reviews suggesting that the vast majority of the prognostic models using PROMs as predictors in oncology lack external validation [17, 63, 67]. External validation is imperative because good performance during development is not guaranteed when transporting a model to other settings [68]. This lack of external validation limits the clinical implementation and application of PROM-based prognostic models, leaving their potential value for enhancing clinical decision-making untapped.

Previous reviews highlighted the significance of PROMs in the prediction of the overall survival in patients with cancer [17, 63]. Although we did not limit the present review to survival as the predicted outcome, we only identified one study that predicted self-reported health status one year after surgery in patients with NSCLC [47]. Survival is not the only outcome considered relevant when making treatment decisions in patients with NSCLC [69]. This warrants an increased effort to predict a wider range of outcomes in addition to survival, such as treatment response [70], treatment toxicity [71], and early treatment discontinuation [72], which in turn would better support patients and doctors in making complex treatment decisions.

This scoping review has several limitations. Firstly, our search strategy needed to balance sensitivity (or comprehensiveness) against specificity (or precision). So, although we had a comprehensive search strategy based on previously published reviews, some relevant studies may have been missed. further studies of interest may have been missed because we excluded those reported as conference abstracts, in non-peer reviewed journals or in a non-English language. Secondly, this review did not distinguish between cancer stages, despite prognosis of patients with early/locally advanced stages NSCLC being different to that of patients with advanced stages [73, 74]. Therefore, we cannot draw conclusions on if and how the prognostic value of PROMs has been investigated differs between stages of NSCLC.

## Conclusion

This scoping review identified an emerging body of research how PROMs have been used as a prognostic factor for predicting a range of clinical outcomes in patients with NSCLC but the methodological quality of this research is poor. This warrants more robust studies investigating the prognostic value of PROMs, in particular for predicting outcomes other than survival. This will enable further development of PROM-based prediction models to support clinical decision making in NSCLC.

## Electronic supplementary material

Below is the link to the electronic supplementary material.

Supplementary Material 1

## Data Availability

The data supporting the conclusions of this study are included within this article and its supplementary information files.

## Abbreviations

**EORTC QLQ-C30**: The European Organization for Research and Treatment of Cancer Quality of Life Questionnaire – Core 30.
**EORTC QLQ-LC13**: EORTC QLQ-Lung cancer 13.
**HRQOL**: Health-related quality of life.
**NSCLC**: Non-small cell lung cancer.
**PROBAST**: Prediction model risk of bias assessment tool.
**PROMs**: Patient-reported outcome measures.
**QUIPS**: Quality in prognosis studies.
