# Supplementary material for: Prognostic value of patient-reported outcome measures (PROMs) in adults with non-small cell Lung Cancer: a scoping review
Source: BMC Cancer. 2022 Oct 19;22:1076. doi: 10.1186/s12885-022-10151-z (PMC9580146; doi:10.1186/s12885-022-10151-z)
Supplement: Supplementary file 1 — Supplementary Material 1 [file 12885_2022_10151_MOESM1_ESM.pdf]

Appendix 1. Search syntax

Medline and Embase via Ovid online

	**NSCLC** [21]
1.	exp Lung Neoplasms/
2.	exp Carcinoma, Non-Small-Cell Lung/
3.	nsclc.tw.
4.	(lung$ or pulmonary or bronchus or bronchogenic or bronchial or bronchoalveolar or alveolar).tw.
5.	(non small cell or non-oat cell).tw.
6.	(cancer or carcinoma$ or neoplasm$ or malignan$ or tumo?r).tw.
7.	4 and 5 and 6
8.	1 or 2 or 3 or 7
	**PROMs** [22]
9.	"Outcome Assessment (Health Care)"/
10.	"Quality of Life"/
11.	(quality of life or QOL).mp.
12.	10 or 11
13.	exp Questionnaires/
14.	(questionnaire* or interview* or self-report* or measure* or instrument* or scale* or tool* or construct*).mp.
15.	13 or 14
16.	12 and 15
17.	(patient* adj5 (report* or relate*) adj5 (outcome* or measure*)).mp.
18.	9 or 16 or 17
	**Prediction and prognosis** [23]
19.	Validat$ OR Predict$.ti. OR Decision Support Techniques/ OR Rule$ OR Predictive Value of Tests/
20.	Predict$ AND (Clinical$ OR Identif$)
21.	19 OR 20
22.	8 and 18 and 21
23.	limit 22 to human
24.	limit 23 to English language
25.	remove duplicates from 24
26.	limit 25 to yr="2011 -Current"

CINAHL Plus

1. MH Lung Neoplasms
2. MH Carcinoma, Non-Small-Cell Lung
3. TX nsclc
4. TX lung* or pulmonary or bronchus or bronchogenic or bronchial or bronchoalveolar or alveolar
5. TX non small cell or non-oat cell
6. TX cancer or carcinoma* or neoplasm* or malignan* or tumo?r
7. S4 and S5 and S6
8. S1 or S2 or S3 or S7
9. MW Outcome Assessment (Health Care)
10. MW Quality of Life
11. quality of life or QOL
12. S10 or S11
13. MH Questionnaires
14. questionnaire* or interview* or self-report* or measure* or instrument* or scale* or tool* or construct*
15. S13 or S14
16. S12 and S15
17. patient* report* outcome* or measure*
18. S9 or S16 or S17
19. (Validat* OR Predict*).ti. OR (Decision Support Techniques) OR (Rule* OR Predictive Value of Tests)
20. Predict* AND (Clinical* OR Identif*)
21. S19 OR S20
22. S8 and S18 and S21

Scopus

((TITLE-ABS-KEY ("non-small cell lung cancer")) OR (TITLE-ABS-KEY (nsclc)) OR (TITLE-ABS-KEY ("non-small cell lung carcinoma")) OR (TITLE-ABS-KEY ("non-small cell lung neoplasm")) OR (TITLE-ABS-KEY ("non-small cell lung tumor")) OR (TITLE-ABS-KEY ("non-small cell lung malignancy"))) AND ((TITLE-ABS-KEY (symptom)) OR (TITLE-ABS-KEY ("quality of life")) OR (TITLE-ABS-KEY ("patient-reported outcome"))) AND ((TITLE-ABS-KEY (predict)) OR (TITLE-ABS-KEY (prognosis)) OR (TITLE-ABS-KEY (prognostic))) AND PUBYEAR > 2010 AND NOT (INDEX (medline) OR INDEX (embase)) AND (LIMIT-TO (LANGUAGE , "English"))

Appendix 2. Inclusion and exclusion criteria

Criterion	*Inclusion*	*Exclusion*
**Population**	Adults aged ≥18 years diagnosed with NSCLC.<br>We included studies with mixed samples (e.g. patients with different types of cancer) if ≥50% were patients with NSCLC or if results are reported separately for NSCLC.	Adults diagnosed with other types of (lung) cancer; adult with a high risk of NSCLC but not yet diagnosed
**Concept**	Any generic, cancer-specific or lung cancer-specific patient-reported outcome measures or their components collected in any setting (e.g. routine care, clinical trials)	Patient-reported experience of/satisfaction with care; clinician-reported outcomes
**Context**	Prognostic prediction of any future outcome at individual patient-level	Diagnostic prediction of having a certain condition at the time of prediction
**Study and publication type**	Articles or full conference papers reporting the findings of association studies or prediction model development or validation studies.	Pre-prints, protocols, reviews, commentaries, editorials, conference abstracts and non-peer-reviewed articles.
**Publication date**	From 2011 to current [a)]	Before 2011
**Language**	English	Other languages

a) NSCLC survival has improved because of recent advances in treatment [24–26]. For this reason, we limited the publication date to the last ten years

Appendix 3. Definition of data extraction items

Main category	Definition	Sub-categories
**Characteristics of the study**		
Reference citation	Identify the study from the studies included in the review	
Years of publication	Year of publication	
Country(ies)	Where the study was conducted	**Free text**
Aim of study	the aim of each included study	**Free text**
Type of population	The characteristics of the population	**NSCLC only**, **mixed population** (with the percentage of NSCLC patients accounted for the whole)
Study design	What type of prognostic study each study is.	**Prognostic factor study -** To identify candidate prognostic factors (prognostic markers /determinants);<br><br>**Prediction model development -** To determine predictors (prognostic markers/determinants) of an outcome. And give the probability of an outcome.<br><br>**Prediction model external validation -** To determine if the prediction model predicts well in external populations.
Data source	Where the data in the included study was from	**Clinical trial** (including secondary analyses); **Observational study** (including cohort, case-control studies); **Routinely collected data** (e.g., data derived from patient records)
Median follow-up	The length of time from baseline to half of the patients in a group of patients developed a certain outcome.	
Sample size	Number of participants included in the analysis	
**Characteristics of PROMs as predictors**		

Full name of PROMs	The full name of the PROMs included in the prognostic analysis	**Free text**
Abbreviated name of PROMs	The well-acknowledged abbreviated name of the PROMs above.	**Free text**
Construct PROM aims to capture	What patient-reported aspects of the PROMs were measured [75].	**Health-related quality of life** – refers to a multidimensional construct encompassing physical, social, and emotional well-being associated with illness and its treatment.<br><br>**Functional status** - refers to a patient's ability to perform both basic and more advanced (instrumental) activities of daily life**;** **Symptoms and symptom burden** – symptoms and their presence and intensity
Classification of PROMs	What level the PROMs were measuring.	**Generic -** measure the wellbeing of all types of patients, regardless of their illness or disorder;<br><br>**Cancer-specific –** only measure the wellbeing of patients with cancer;<br><br>**Lung cancer-specific –** only measure the wellbeing of patients with lung cancer.
Cross-sectional vs change in score	If the included score is cross-sectional was used or a change in score over time	**Cross-sectional PROMs score**; **Change in scores**<br><br>(for change in scores, describe how this was calculated)
PROMs included in the model	If single item scores, subdomain scores or overall summary scores of the PROMs were used in the model	**Single item scores –** score for each item;<br><br>**Subdomain scores –** consist of a group of scores measuring for the same aspect of wellbeing;<br><br>**Overall summary scores –** scores summarised all the items.
Time points of PROMs collection	At what time point PROMs scores included in the model had been collected	**Free text**
**Characteristics of outcome(s)**		

Number of outcomes	The total number of outcomes assessed in the study including both primary and secondary outcomes	
Name and description of primary outcome	The primary outcome(s) is assessed in the prognostic factor study or in the prediction model.	**Free text**
Type of primary outcome	Classified as per the lung cancer standard set of outcomes [76]	**Acute complications of treatment** (e.g., major surgical complications, major radiation complications);<br><br>**Degree of health** (e.g., performance status, quality of life),<br><br>**Survival** (progression-free survival, overall survival); **Quality of death** (e.g., place of death, duration of time spent in hospital at end of life), **Other** (e.g., time from diagnosis to treatment)
Name and description of secondary outcome	The secondary outcome(s) assessed in the prognostic factor study or in the prediction model.	**Free text**
Type of secondary outcome	Classified as per the lung cancer standard set of outcomes [76].	**Acute complications of treatment** (e.g., major surgical complications, major radiation complications);<br><br>**Degree of health** (e.g., performance status, quality of life),<br><br>**Survival** (progression-free survival, overall survival); **Quality of death** (e.g., place of death, duration of time spent in hospital at end of life), **Other** (e.g., time from diagnosis to treatment)
Time points of outcome assessment	Time points at which outcomes were assessed.	**At the time of occurrence** (e.g., death); **At the time of clinic visit/follow-up** (need to specify the frequency of visits/follow-ups. e.g., treatment discontinuation)
Maximum length of observation	The maximum time between the end of observation and baseline.	**Free text**
**Statistical methods**		
Types of predictive modelling techniques	The statistical techniques are used to evaluate the association between candidate prognostic factors and outcomes or in the prediction model.	**e.g., Cox proportional hazard model**;<br><br>**Decision tree;**

		**Support vector machine;**<br>**Naïve bayes**
Uni/multivariable	If there are single or multiple independent variables/predictors in a model.	**Univariable –** only one independent variable/predictor in one model<br>**Multivariable –** multiple independent variables/predictors in one model
Uni/multivariate	If there is single or multiple dependent variables/outcomes in a model.	**Univariate –** only one dependent variable/outcome in one model<br>**Multivariate –** multiple dependent variables/outcomes in one model (i.e. simultaneous prediction of multiple outcomes)
Selection of predictors/independent variables	How the studies selected the predictors/independent variables in the model.	**Free text**
Other predictors in the prognostic models	Describe other predictors included in the prognostic model.	**Free text**
Type of confounders/other predictors	To classify variables other than PROMs in the adjusted analyses or in the prediction model	**Demographics**, **treatment**, **performance status, genetic biomarkers, complications, tumour characteristics, comorbidities, physiological measurements, others**

Appendix 4. Characteristics of the included studies

Reference citation	Years of publication	Country(ies) /Region(s)	Aim of study	Type of population	Study design	Data source	Sample size
Agarwal et al	2017	India	To ascertain if the patient reported functional status as ascertained using the EORTC QLQ C30 provides additional prognostic information over and above KPS.	NSCLC only	Prediction model development	Observational study	140
Arraras et al	2016	Spain	To study the Quality of Life of Spanish advanced NSCLC patients receiving platinum-doublet chemotherapy, compares our results with those from studies from other cultural areas, and identifies factors associated with global QL and survival prognostic variables.	NSCLC only	Prognostic factor study	Observational study	39
Arrieta et al	2013	Mexico	To evaluate the association of depression and anxiety on HRQL, treatment adherence, and prognosis in patients with non-small cell lung cancer (NSCLC).	NSCLC only	Prognostic factor study	Observational study	54
Barney et al	2011	USA	To test whether adding various PROs to models containing established prognostic factors would improve the prediction of survival in patients with advanced non-small cell lung cancer (NSCLC)	NSCLC only	Prognostic factor study	Observational study	90
Braun et al	2011	USA	To investigate whether QoL can predict survival in non-small cell lung cancer patients treated at a community hospital comprehensive cancer centre.	NSCLC only	Prognostic factor study	Observational study	1194
Brunelli et al	2013	Italy	To develop a survival aggregate score, including objective and subjective patient-based parameters, and assess its prognostic role after major anatomic resection for NSCLC.	NSCLC only	Prediction model development	Observational study	245

Ediebah et al	2014	Belgium, Sweden, Netherlands, Germany, Canada, Australia, USA	To investigated whether changes in HRQoL scores from baseline over time were associated with survival, independent of baseline HRQoL scores, in patients with advanced NSCLC.	NSCLC only	Prognostic factor study	Clinical trial	391
Eser et al	2016	Turkey	To examine the relationship of Health related quality of life measured by EORTC QLQc30, QLQ-LC13; FACT-L, LCSS, Eq5D) with survival in advanced lung cancer patients	Mixed population - 60.1% NSCLC	Prognostic factor study	Observational study	299
Fernando et al	2015	USA	To (1) compare SR alone with SR with brachytherapy (SRB) for high-risk operable patients with early-stage non–small cell lung cancer and (2) measure longitudinal QOL, and self-reported functional health status.	NSCLC only	Prognostic factor study	Clinical trial	212
Fiteni et al	2016	France	To investigate whether the HRQOL score is a prognostic factor for overall survival in elderly patients with advanced NSCLC.	NSCLC only	Prognostic factor study	Clinical trial	393
Friis et al	2021	Denmark	To explore the prognostic value of PRO measures at disease progression and the changes in PRO measures between treatment start (baseline) and disease progression.	Mixed population - 76.6% NSCLC	Prognostic factor study	Routinely collected data	94
Greer et al	2014	USA	To expand on prior research concerning cognitive awareness of terminal illness by examining the longitudinal course and correlates of patients' perceptions of their health status as well as the association between perceived health status and survival.	NSCLC only	Prognostic factor study	Clinical trial	151
Gupta et al	2012	USA	To investigated whether pretreatment quality of life parameters as well as changes in quality of life scores from baseline until 3 months after treatment could predict survival in patients with stages III–IV non-small cell lung cancer	NSCLC only	Prognostic factor study	Observational study	430

Hopkins et al	2020	Australia	To evaluate the association between pre-treatment PROs and survival outcomes of patients with advanced NSCLC treated with atezolizumab	NSCLC only	Prognostic factor study	Clinical trial	1426
Jeon et al	2020	Korea	To investigate the prognostic value of the FACT-G in the survival of advanced NSCLC patients treated with Korean medicine	NSCLC only	Prognostic factor study	Routinely collected data	165
Kerstjens et al	2019	Netherlands	To investigate the prognostic value of the DT when combined with sociodemographic and clinical predictors to assess one-year survival in patients with lung cancer.	Mixed population - 80% NSCLC	Prognostic factor study	Clinical trial	97
Kobayashi et al	2021	Japan	To investigate the predictors of postoperative QOL using the EQ-5D VAS and EORTC GHS scores in patients who undergo lung resection for lung cancer.	Mixed population - 97.8% NSCLC	Prognostic factor study	Observational study	223
Lemonnier et al	2014	France	To investigate whether HRQoL after the initial treatment still plays a role in predicting survival in patients with NSCLC.	NSCLC only	Prognostic factor study	Observational study	230
Li et al	2012	Taiwan	To examine the relationship of EORTC QLQ-C30 and QLQ-LC13 and survival in patients with NSCLC undergoing different treatments.	NSCLC only	Prognostic factor study	Observational study	312 (non-surgery model)
Möller et al	2012	Sweden	To analyse the association between changes in quality of life and survival after lung cancer surgery	Mixed population - 80.9% NSCLC	Prognostic factor study	Observational study	141

Movsas et al	2016	USA & Canada	The primary QOL hypothesis predicted a clinically meaningful decline in quality of life (QOL) via the Functional Assessment of Cancer Therapy (FACT)–Lung Cancer Subscale (LCS) in the high-dose RT arm at 3 months	NSCLC only	Prognostic factor study	Clinical trial	302
O'Mahony et al	2016	USA	To assess whether the LCSS score correlates with the PPS score and serial weight change in the prediction of survival in-patients with advanced NSCLC with ECOG score ≥ 1.	NSCLC only	Prognostic factor study	Observational study	62
Pinheiro et al	2018	USA	To (1)investigate associations between HRQOL changes and OS and (2) identify the best HRQOL assessment time point to predict OS	Mixed population - 90%	Prognostic factor study	Routinely collected data	535
Pinheiro et al	2017	USA	To determine the association between pre-lung cancer diagnosis HRQOL and OS	Mixed population - 85% NSCLC	Prognostic factor study	Observational study	6290
Pompili et al	2013	Italy	To assess the prognostic role of Baseline quality of life (QoL) in patients operated on for early-stage non-small-cell lung cancer (NSCLC)	NSCLC only	Prognostic factor study	Observational study	131
Schild et al	2015	USA	To evaluate the outcome of a large patient cohort to identify their Baseline prognostic factors and created a scoring system that can stratify patients into groups with distinctly different outcomes.	NSCLC only	Prediction model development	Routinely collected data	1274
Sim et al	2020	Korea	To evaluate the major roles of health-related quality of life (HRQOL) in a 5-year lung cancer survival prediction model using machine learning techniques.	Not reported	Prediction model development	Routinely collected data	809

Sloan et al	2012	USA	(1) To confirm the prognostic value of QOL at the time of lung cancer diagnosis in predicting survival among patients with lung cancer and (2) to identify a patient profile associated with poor QOL at the time of diagnosis of lung cancer.	NSCLC only	Prognostic factor study	Observational study	2442
Spigel et al	2015	USA	An additional secondary end point focused on QoL and on evaluating differences in patient-reported outcomes, as assessed by the FACT-G, FACT-L, and FACT/Gynecologic Oncology Group-Neurotoxicity (FACT-Ntx) instruments; these results are presented here as are investigator-reported toxicity scores and associated resource use. Post hoc analyses that examined prognostic factors for OS are also reported	NSCLC only	Prognostic factor study	Clinical trial	885
Stene et al	2015	Norway	To explored whether change muscle is an independent prognostic factor for survival	NSCLC only	Prognostic factor study	Observational study	35
Yun et al	2016	Korea	To evaluate the prognostic value of QOL, which provides information on the likely course of cancer mortality by predicting survival among patients with lung cancer after cancer treatment completion	NSCLC only	Prognostic factor study	Observational study	809

Appendix 5. Characteristics of PROMs as predictors

Reference citation	Full name of PROMs	Abbreviated name of PROMs	Aspect of PROMs measuring	Classification of PROMs	Single PROMs score vs change in score	Form of PROMs included in the model	Time points of PROMs collection
Agarwal et al	European Organization for Research and Treatment of Cancer Quality of Life Questionnaire Core 30;	EORTC QLQ-C30	Health-related quality of life	Cancer specific	Single PROMs score	Separate items	Baseline
Arraras et al	European Organization for Research and Treatment of Cancer Quality of Life Questionnaire Core 30 and Lung Cancer 13	EORTC QLQ-C30; EORTC QLQ-LC13	Health-related quality of life	Cancer-specific; Lung cancer-specific	Single PROMs score	Subdomains	Baseline
Arrieta et al	European Organization for Research and Treatment of Cancer Quality of Life Questionnaire Core 30 and Lung Cancer 13; hospital anxiety and depression scale	EORTC QLQ-C30; EORTC QLQ-LC13; HADS	Health-related quality of life	Cancer-specific; Lung cancer-specific	Single PROMs score	Summary scores	Baseline
Barney et al	12-item Short Form Health Survey and M. D. Anderson Symptom Inventory - Lung Cancer	SF-12, MDASI-LC	Health-related quality of life; Symptoms and symptom burden	Generic; Lung cancer-specific	Single PROMs score	Subdomains	Baseline

Braun et al	European Organization for Research and Treatment of Cancer Quality of Life Questionnaire Core 30	EORTC QLQ-C30	Health-related quality of life	Cancer specific	Single PROMs score	Subdomains	Baseline
Brunelli et al	Short-Form 36v2	SF36v2	Health-related quality of life	Generic	Single PROMs score	Subdomains	Baseline
Ediebah et al	European Organization for Research and Treatment of Cancer Quality of Life Questionnaire Core 30 and Lung Cancer 13	EORTC QLQ-C30; EORTC QLQ-LC13	Health-related quality of life; Symptoms and symptom burden	Cancer-specific; Lung cancer-specific	Analysis 1: baseline Analysis 2: change in score (baseline - cycle 1, baseline - cycle 2, baseline - cycle 3)	Subdomains	Analysis 1: baseline (before treatment); Analysis 2: baseline and at the end of 1st, 2nd and 3rd cycle of treatment
Eser et al	European Organization for Research and Treatment of Cancer Quality of Life Questionnaire Core 30 and Lung Cancer 13; Functional Assessment of Cancer Therapy - Lung; Lung Cancer Symptom Scale; EuroQol-5 Dimension	EORTC QLQ-C30; EORTC QLQ-LC13; FACT-L; LCSS; EQ-5D	Health-related quality of life; Symptoms and symptom burden	Generic; Cancer-specific; Lung cancer-specific	Single PROMs score	Subdomains and separate items	Baseline

Fernando et al	the 36-item Short-Form Health Survey<br>The University of California San Diego Shortness of Breath Questionnaire	Analysis 1:SF-36; Analysis 2: SOBQ	Health-related quality of life; functional status	Generic	Analysis 1: change in scores (10-points decline in SF-36 score at 3 and 12 months)<br>Analysis 2: change in scores (10-points decline in SOBQ)	Summary scores	Baseline
Fiteni et al	European Organization for Research and Treatment of Cancer Quality of Life Questionnaire Core 30	EORTC QLQ-C30	Health-related quality of life	Cancer specific	Single PROMs score	Subdomains	Baseline
Friis et al	European Organization for Research and Treatment of Cancer Quality of Life Questionnaire Core 30 and Lung Cancer 13	EORTC QLQ-C30; EORTC QLQ-LC13	Health-related quality of life; Symptoms and symptom burden	Cancer-specific; Lung cancer-specific	Single PROMs score; change in score (score at disease progression - score at treatment start)	Separate items	Baseline (treatment start); Disease progression (median 5.9 months; IQR, 3.0-8.4)
Greer et al	Functional Assessment of Cancer Therapy - Lung, Hospital Anxiety and Depression Scale, Patient Health Questionnaire-9	FACT-L, HADS, PHQ-9	Health-related quality of life; Symptoms and symptom burden	Generic; Lung cancer-specific	Change in score (time-varying predictors)	Subdomains	Baseline (at enrolment), 12, 18, and 24 weeks
Gupta et al	European Organization for Research and Treatment of Cancer Quality of Life Questionnaire	EORTC QLQ-C30; EORTC QLQ-LC13	Health-related quality of life; Symptoms and symptom burden	Cancer-specific; Lung cancer-specific	Single PROMs score, Change in score (3 months - baseline)	Subdomains	Baseline, 3 months

	Core 30 and Lung Cancer 13						
Hopkins et al	European Organization for Research and Treatment of Cancer Quality of Life Questionnaire Core 30 and Lung Cancer 13	EORTC QLQ-C30; EORTC QLQ-LC13	Health-related quality of life; Symptoms and symptom burden	Cancer-specific; Lung cancer-specific	Single PROMs score	Separate items	Baseline
Jeon et al	Functional Assessment of Cancer Therapy-General	FACT-G	Health-related quality of life	Generic	Single PROMs score	Subdomains and summary scores	Within 1 month of the first visit day for treatment
Kerstjens et al	Distress Thermometer	DT	Health-related quality of life	Generic	Single PROMs score	Summary scores	Baseline
Kobayashi et al	EuroQol-5 Dimension 5 levels and visual analogue scale; European Organization for Research and Treatment of Cancer Quality of Life Questionnaire Core 30	EQ-5D-5L; EQ-5D VAS; EORTC QLQ-C30	Health-related quality of life	Generic	Single PROMs score	Separate items	Baseline

Lemonnier et al	the 36-item Short-Form Health Survey; European Organization for Research and Treatment of Cancer Quality of Life Questionnaire Core 30	SF-36; EORTC QLQ-C30	Health-related quality of life	Generic; Cancer-specific	Single PROMs score	Subdomains	3 months after diagnosis
Li et al	European Organization for Research and Treatment of Cancer Quality of Life Questionnaire Core 30 and Lung Cancer 13	EORTC QLQ-C30; EORTC QLQ-LC13	Health-related quality of life; Symptoms and symptom burden	Cancer-specific; Lung cancer-specific	Single PROMs score	Subdomains	Baseline
Möller et al	the 36-item Short-Form	SF-36	Health-related quality of life	Generic	Model 1: Single PROMs score; Model 2: Change in scores (the baseline SF-36 subscale score was subtracted from the 6-month scores)	Subdomains and summary scores	Model 1: baseline; Model 2: baseline and 6 month after lung cancer surgery
Movsas et al	Functional Assessment of Cancer Therapy - Lung	FACT-L	Health-related quality of life	Lung cancer-specific	Single PROMs score	Summary scores	Baseline
O'Mahony et al	Lung Cancer Symptom Scale	LCSS	Symptoms and symptom burden	Lung cancer-specific	Single PROMs score	Summary scores	Baseline (at enrollment)

Pinheiro et al	Short-Form 36; Katz's Activities of Daily Living	SF-36; ADL	Health-related quality of life	Generic	Single PROMs score; change in score (prediagnosis score - postdiagnosis score)	Subdomains and summary scores	Prediagnosis (median time from survey to diagnosis = 13 months); Postdiagnosis (median time from diagnosis to post diagnosis survey
Pinheiro et al	the 36-item Short-Form Health Survey	SF-36	Health-related quality of life	Generic	Single PROMs score	Subdomains and summary scores	Baseline
Pompili et al	Short-Form 36v2	SF36v2	Health-related quality of life	Generic	Single PROMs score	Subdomains	Baseline
Schild et al	Lung Cancer Symptom Scale	LCSS	Symptoms and symptom burden	Lung cancer-specific	Single PROMs score	One item (Global QOL)	Baseline
Sim et al	European Organization for Research and Treatment of Cancer Quality of Life Questionnaire Core 30; Hospital Anxiety and Depression Scale; Posttraumatic Growth Inventory	EORTC QLQ-C30; HADS; PTGI	Health-related quality of life; Symptoms and symptom burden	Generic; Cancer-specific	Single PROMs score	Separate items	Baseline

Sloan et al	Lung Cancer Symptom Scale	LCSS	Health-related quality of life	Lung cancer-specific	Single PROMs score	One item (Global QOL)	Baseline
Spigel et al	Functional Assessment of Cancer Therapy - general, Lung and FACT/Gynecologic Oncology Group-Neurotoxicity	FACT-G, FACT-L, and FACT-Ntx	Health-related quality of life	Generic; Lung cancer-specific	Single PROMs score	Summary scores	Baseline
Stene et al	European Organization for Research and Treatment of Cancer Quality of Life Questionnaire Core 30	EORTC QLQ-C30	Health-related quality of life	Cancer specific	Single PROMs score	Summary scores	Not reported
Yun et al	European Organization for Research and Treatment of Cancer Quality of Life Questionnaire Core 30 and lung cancer 13, Hospital Anxiety and Depression Scale, and Posttraumatic Growth Inventory	EORTC QLQ-C30; EORTC QLQ-LC13; HADS; PTGI	Health-related quality of life; Symptoms and symptom burden	Generic; Cancer-specific; lung cancer-specific	Single PROMs score	Subdomains	Baseline

Need to include abbreviations under all tables

Appendix 6. Characteristics of the outcome(s)

Reference citation	Number of outcomes	Name and description of primary outcome	Type of primary outcome	Name and description of secondary outcome	Type of secondary outcome	Time points of outcome assessment	Median follow-up	Maximum length of observation
Agarwal et al	1	Overall survival	Survival	N/A	N/A	At time of occurrence	166 days (95% CI: 108–242 days)	990 days
Arraras et al	1	Overall survival	Survival	N/A	N/A	At time of occurrence	7.45 months	44.63 months
Arrieta et al	1	Overall survival	Survival	N/A	N/A	At time of occurrence	9.6 months	27 months
Barney et al	1	Overall survival	Survival	N/A	N/A	At time of occurrence	8.4 months	Not reported
Braun et al	1	Overall survival	Survival	N/A	N/A	At time of occurrence	8.8 months (95%CI: 8.0-9.5 months)	7 years

Brunelli et al	1	Overall survival	Survival	N/A	N/A	At time of occurrence	37 months	7 years
Ediebah et al	1	Overall survival	Survival	N/A	N/A	At time of occurrence	Not reported	2 years
Eser et al	1	Overall survival	Survival	N/A	N/A	At time of occurrence	8.0 months	22 months
Fernando et al	2	Overall survival	Survival	Recurrence-free survival (the time from randomisation to the first of any recurrence or death from any cause)	Survival	At time of occurrence	4.4 years	5.59 years
Fiteni et al	1	Overall survival	Survival	N/A	N/A	At time of occurrence	30.3 months	45.2 months
Friis et al	1	Overall survival	Survival	N/A	N/A	At time of occurrence	10.5 months	Data extracted from 2014 to 2018

Greer et al	1	Overall survival	Survival	N/A	N/A	At time of occurrence	Not reported	3 years
Gupta et al	1	Overall survival	Survival	N/A	N/A	At time of occurrence	13.1 months (95% CI: 11.1–15.1 months).	Not reported
Hopkins et al	3	Overall survival	Survival	Progression free survival; treatment related adverse events	Survival; acute complications of treatment	At time of occurrence	15 months (95% CI, 15-15)	Not reported
Jeon et al	1	Overall survival	Survival	N/A	N/A	At time of occurrence	10.1 months (95% CI: 8.3–11.7)	11 years
Kerstjens et al	1	Overall survival	Survival	N/A	N/A	At time of occurrence	Not reported	3.5 years

Kobayashi et al	1	EQ-5D VAS and EORTC GHS	Degree of health	N/A	N/A	One year postoperatively	Not reported	1 year
Lemonnier et al	1	Overall survival	Survival	N/A	N/A	At time of occurrence	18.5 months (95% CI, 14 – 27)	8 years
Li et al	1	Overall survival	Survival	N/A	N/A	At time of occurrence	9.82 months	3 years and 5 months
Möller et al	1	Overall survival	Survival	N/A	N/A	At time of occurrence	3.7 years	62 months
Movsas et al	1	Overall survival	Survival	N/A	N/A	At time of occurrence	28.7 months (95% CI 24.1–36.9)	48 months
O'Mahony et al	1	Overall survival	Survival	N/A	N/A	At time of occurrence	180.6 ± 109.4 days	381 days
Pinheiro et al	1	Overall survival	Survival	N/A	N/A	At time of occurrence	Not reported	Not reported

Pinheiro et al	1	Overall survival	Survival	N/A	N/A	At time of occurrence	Not reported	7 years
Pompili et al	1	Overall survival	Survival	N/A	N/A	At time of occurrence	40 months	8 years
Schild et al	1	Overall survival	Survival	N/A	N/A	At time of occurrence	Not reported	11 years
Sim et al	1	Overall survival	Survival	N/A	N/A	At time of occurrence	During the 5-year follow-up, 96 deaths (11.9%) and 713 (89.1%) survivals among the 809 subjects were identified.	5 years
Sloan et al	1	Overall survival	Survival	N/A	N/A	At time of occurrence	QOL > 50: 5.6 years, QOL ≤ 50: 1.5 years	11 years
Spigel et al	1	Overall survival	Survival	N/A	N/A	At time of occurrence	Not reported	3 years and 2 months
Stene et al	1	Overall survival	Survival	N/A	N/A	At time of occurrence	Men: 7.9 months Women: 7.2 months	Not reported
Yun et al	1	Overall survival	Survival	N/A	N/A	At time of occurrence	8.3 ± 2.01 years	4 years

Appendix 7. The statistical methods applied in the included studies.

Reference citation	Types of predictive modelling techniques	Uni/multivariable	Uni/multivariate	Selection of predictors/independent variables	Confounders/other predictors in the prognostic models	Type of confounders/other predictors
Agarwal et al	Cox proportional hazards regression	Multivariable	Univariate	Not reported	Age, gender, number of brain metastases (1–3 or 3 or more), extracranial disease (present/absent), epidermal growth factor receptor (EGFR) mutation (mutated/wild type/not tested)	Demographics; tumour characteristics; genetic biomarker
Arraras et al	Cox proportional hazards regression	Multivariable	Univariate	Not reported	Age and gender	Demographics
Arrieta et al	Cox proportional hazards regression	Multivariable	Univariate	Not reported	Age, gender, smoking history, Karnofsky performance status, clinical stage, histology	Demographics, performance status, tumour characteristics
Barney et al	Cox proportional hazards regression	Multivariable	Univariate	Not reported	Sex, age, previous chemotherapy, and ECOG PS	Demographics, treatment, performance status
Braun et al	Cox proportional hazards regression	Multivariable	Univariate	Select the variables that were significant in the univariable analysis and use forward stepwise selection and block entry method.	Gender, stage at diagnosis, prior treatment history	Demographics, tumour characteristics, treatment

Brunelli et al	Cox proportional hazards regression	Multivariable	Univariate	Select the variables that were significant in the univariable analysis	Age, FEV1 percentage, FEV1/ FVC ratio, DLCO percentage, ECOG score	Demographics, performance status, other
Ediebah et al	Cox proportional hazards regression	Multivariable	Univariate	Stepwise selection	Age, gender, stage of disease (IIIB vs IV), histological subtype and WHO performance status.	Demographics, performance status, tumour characteristics
Eser et al	Cox proportional hazards regression	Multivariable	Univariate	Not reported	Age, gender, clinical stage and comorbidities	Demographics, tumour characteristics, comorbidities
Fernando et al	Cox proportional hazards regression	Analysis 1 (SF-36): Multivariable; Analysis 2 (SOBQ): Univariable	Univariate	Not reported	Analysis 1: Age, gender; Analysis 2: NA	Analysis 1: Demographics; Analysis 2: NA
Fiteni et al	Cox proportional hazards regression	Multivariable	Univariate	HRQOL scores + predictors in a reference risk model	Treatment, performance score, smoking status, histology, mini-mental state; activities of daily living	Smoking status, tumour characteristics, performance status, physiological parameters

Friis et al	Cox proportional hazards regression	Univariable	Univariate	N/A	N/A	N/A
Greer et al	Cox proportional hazards regression	Multivariable	Univariate	Not reported	Age, sex, marital status, ECOG performance status, smoking status, brain metastasis, initial cancer treatment	Demographics, performance status, smoking status, tumour characteristics, treatment
Gupta et al	Cox proportional hazards regression	Multivariable	Univariate	Not reported	Age, gender, stage at diagnosis and prior treatment history	Demographics, tumour characteristics, treatment
Hopkins et al	Cox proportional hazards regression	Multivariable	Univariate	Select the variables that were significant in the univariable analysis	Age, sex, race, smoking history, histology, prior treatments, PD-L1 expression, lactate dehydrogenase levels, derived neutrophil to lymphocyte ratio (dNLR; calculated as neutrophil/[white blood cell−neutrophil count]) and the presence of liver, lung or brain metastases	Demographics, tumour characteristics, treatment, genetic biomarkers, other
Jeon et al	Cox proportional hazards regression	Multivariable	Univariate	Select the variables that were significant in the univariable analysis	Age, sex, smoking history, ECOG-PS, histological type, and presence of extra-thoracic metastasis	Demographics, performance status, tumour characteristics
Kerstjens et al	Cox proportional hazards regression	Multivariable	Univariate	Based on the literature as well as expert opinion and availability of such predictors in clinical settings.	Gender; Performance status; Disease stage; The Charlson age-adjusted comorbidity index; Tumour histology.	Demographics, performance status, tumour characteristics, comorbidities

Kobayashi et al	Linear regression	Multivariable	Univariate	Select the variables that were significant in the univariable analysis	Age, sex, living alone, less invasive surgical approach, sublobar resection as lung resection mode, adjuvant therapy, and postoperative adverse events	Demographics, treatment, other
Lemonnier et al	Cox proportional hazards regression	Multivariable	Univariate	Not reported	Age, sex, cancer stage, and treatment	Demographics, tumour characteristics, treatment
Li et al	Cox proportional hazards regression	Multivariable	Univariate	Select the variables that were significant in the univariable analysis	Age, gender, cancer duration, type of cancer, cancer clinical stage of TNM	Demographics, tumour characteristics
Möller et al	Cox proportional hazards regression	Multivariable	Univariate	Not reported	Model 1: age, gender, comorbidities, extent of resection, tumour stage, smoking status, and postoperative complications<br>Model 2: age, gender, comorbidities, extent of resection, tumour stage, smoking status, postoperative complications, and corresponding baseline SF-36 summary or subscale score.	Model 1: Demographics, tumour characteristics, smoking status, complications;<br>Model 2: Demographics, tumour characteristics, smoking status, complications, PROMs.
Movsas et al	Cox proportional hazards regression	Multivariable	Univariate	Not reported	Radiation level, cetuximab assignment, planning target volume, Volume of heart receiving 5 Gy or more radiation	Treatment
O'Mahony et al	Cox proportional hazards regression	Multivariable	Univariate	Not reported	Age, baseline weight, baseline ECOG score, baseline Palliative Performance Scale (PPS) score	Demographics, tumour characteristics, physiological parameters

Pinheiro et al	Cox proportional hazards regression	Multivariable	Univariate	Based on clinical knowledge	Pre-existing health conditions (e.g., hypertension, cardiovascular disease, stroke, emphysema, asthma, chronic obstructive pulmonary disease, gastrointestinal disease, arthritis, sciatica, and diabetes), education, smoking, marital status, age at diagnosis, sex, gender, cancer stage (local, regional, distant), subtype(NSCLC or SCLC), and treatments (surgery and radiation), survey mode of administration (paper or telephone) as well as pre- and post-diagnosis MHOS completion by proxy.	Demographics, treatment, comorbidities, tumour characteristics, other
Pinheiro et al	Cox proportional hazards regression	Multivariable	Univariate	Model covariates were consistent with the previous SEER-MHOS studies	Age at diagnosis, sex, and race, whether or not the MHOS was completed by a proxy, cancer stage at diagnosis, whether the lung cancer was NSCLC or SCLC, and treatments received	Demographics, survey administration methods, tumour characteristics, treatment
Pompili et al	Cox proportional hazards regression	Multivariable	Univariate	Select the variables that were significant in the univariable analysis	Age, FEV1%, ECOG performance status	Demographics, performance status, other
Schild et al	Cox proportional hazards regression	Multivariable	Univariate	Select the variables that were significant in the univariable analysis	Age, sex, ECOG performance status, smoking cessation, tumour size, regional nodal involvement, distant metastasis	Demographics, performance status, tumour characteristics
Sim et al	Decision tree; Logistic regression; Bagging; Adaptive boosting; Random forest	Multivariable	Univariate	Candidate variables that met both the literature review evidence level and statistical significance based on univariate analyses from a previous study were selected. An automatic variable selection method was applied.	Age; Gender; Household income; Cancer stage; Local invasion of tumour; Regional lymph node metastasis; BMI before the operation	Demographics; tumour characteristics, physiological parameters

Sloan et al	Cox proportional hazards regression	Multivariable	Univariate	Based on clinical knowledge	age, sex, race, comorbidities, employment status, marital status, years of education, pack years, never, former, recent quitter, still smoking, histology, stage, grade, chemotherapy, radiation, surgery	Demographics, smoking status, tumour characteristics, comorbidities, treatment
Spigel et al	Cox proportional hazards regression	Multivariable	Univariate	Not reported	Treatment, stratification factors (i.e., stage of disease, ECOG PS, sex, and measureable disease),	Demographics, treatment, tumour characteristics, performance status
Stene et al	Cox proportional hazards regression	Multivariable	Univariate	Not reported	Gender, performance status, stage of cancer, treatment regime, treatment response, BMI, WL 3 months pre-study, C-reactive protein, albumin, baseline sarcopenia, SMCA change, appetite loss	Demographics, tumour characteristics, performance status, other
Yun et al	Cox proportional hazards regression	Multivariable	Univariate	Select the variables that were significant in the univariable analysis and use backward elimination	Age at diagnosis, sex, cancer stage, BMI after surgery.	Demographics, tumour characteristics, physiological parameters

Appendix 8. Domains assessed for risk of bias using the QUIPS tool.

Study	Study participation	Study attrition	Prognostic factor	Outcome measurement	Study confounding	Statistical analysis and reporting	Overall
Arraras 2016	High	Moderate	High	Low	Moderate	Moderate	High
Arrieta 2013	Moderate	High	Moderate	Low	Moderate	Moderate	High
Barney 2011	Low	High	Moderate	Low	Moderate	High	High
Braun 2011	Low	Low	Low	Low	Moderate	Low	Low
Ediebah 2014	Low	Low	Moderate	Low	Moderate	Low	Moderate
Eser 2016	Moderate	Low	Moderate	Low	Moderate	Low	High
Fernando 2015	Low	Moderate	Moderate	Low	Moderate	Moderate	High
Fiteni 2016	Low	Moderate	Moderate	Low	High	Low	High
Friis 2021	Moderate	High	Moderate	Low	High	Moderate	High
Greer 2014	High	Low	Low	Low	Low	Moderate	High
Gupta 2012	Low	Low	Low	Low	Moderate	Moderate	High
Hopkins 2020	Moderate	High	Low	Low	Moderate	Moderate	High
Jeon 2020	Low	Low	Moderate	Low	Moderate	Moderate	High
Kerstjens 2019	Moderate	High	Moderate	Low	Moderate	Moderate	High
Kobayashi 2021	Low	High	Moderate	Low	Moderate	Moderate	High
Lemonnier 2014	Moderate	High	Moderate	Low	Moderate	Moderate	High
Li 2012	Low	High	Moderate	Low	Moderate	Moderate	High
Möller 2012	Low	Moderate	Moderate	Low	Moderate	Low	High
Movsas 2016	Low	Moderate	Moderate	Low	Moderate	Low	High
O'Mahony 2016	Moderate	High	Moderate	Low	Moderate	Moderate	High
Pinheiro 2018	Low	Low	Moderate	Low	Moderate	Low	Moderate
Pinheiro 2017	Moderate	High	Moderate	Low	Moderate	Low	High
Pompili 2013	Moderate	High	Moderate	Low	Moderate	High	High
Sloan 2012	Low	Low	Moderate	Low	Moderate	Low	Moderate
Spigel 2015	Low	Moderate	High	Low	Moderate	High	High
Stene 2014	Moderate	Low	Moderate	Low	Moderate	Low	High
Yun 2016	Moderate	High	Moderate	Low	Moderate	Low	High

Appendix 9. Preferred Reporting Items for Systematic reviews and Meta-Analyses extension for Scoping Reviews (PRISMA-ScR) Checklist

SECTION	ITEM	PRISMA-ScR CHECKLIST ITEM	REPORTED ON PAGE #
**TITLE**			
Title	1	Identify the report as a scoping review.	1
**ABSTRACT**			
Structured summary	2	Provide a structured summary that includes (as applicable): background, objectives, eligibility criteria, sources of evidence, charting methods, results, and conclusions that relate to the review questions and objectives.	2
**INTRODUCTION**			
Rationale	3	Describe the rationale for the review in the context of what is already known. Explain why the review questions/objectives lend themselves to a scoping review approach.	3-4
Objectives	4	Provide an explicit statement of the questions and objectives being addressed with reference to their key elements (e.g., population or participants, concepts, and context) or other relevant key elements used to conceptualize the review questions and/or objectives.	4
**METHODS**			
Protocol and registration	5	Indicate whether a review protocol exists; state if and where it can be accessed (e.g., a Web address); and if available, provide registration information, including the registration number.	N/A
Eligibility criteria	6	Specify characteristics of the sources of evidence used as eligibility criteria (e.g., years considered, language, and publication status), and provide a rationale.	4
Information sources*	7	Describe all information sources in the search (e.g., databases with dates of coverage and contact with authors to identify additional sources), as well as the date the most recent search was executed.	4
Search	8	Present the full electronic search strategy for at least 1 database, including any limits used, such that it could be repeated.	Supp. 1-2

SECTION	ITEM	PRISMA-ScR CHECKLIST ITEM	REPORTED ON PAGE #
Selection of sources of evidence†	9	State the process for selecting sources of evidence (i.e., screening and eligibility) included in the scoping review.	5
Data charting process‡	10	Describe the methods of charting data from the included sources of evidence (e.g., calibrated forms or forms that have been tested by the team before their use, and whether data charting was done independently or in duplicate) and any processes for obtaining and confirming data from investigators.	5
Data items	11	List and define all variables for which data were sought and any assumptions and simplifications made.	Supp. 4-7
Critical appraisal of individual sources of evidence§	12	If done, provide a rationale for conducting a critical appraisal of included sources of evidence; describe the methods used and how this information was used in any data synthesis (if appropriate).	5-6
Synthesis of results	13	Describe the methods of handling and summarizing the data that were charted.	5
**RESULTS**			
Selection of sources of evidence	14	Give numbers of sources of evidence screened, assessed for eligibility, and included in the review, with reasons for exclusions at each stage, ideally using a flow diagram.	7
Characteristics of sources of evidence	15	For each source of evidence, present characteristics for which data were charted and provide the citations.	8-11
Critical appraisal within sources of evidence	16	If done, present data on critical appraisal of included sources of evidence (see item 12).	12-13
Results of individual sources of evidence	17	For each included source of evidence, present the relevant data that were charted that relate to the review questions and objectives.	Supp. 8-30
Synthesis of results	18	Summarize and/or present the charting results as they relate to the review questions and objectives.	8-13
**DISCUSSION**			
Summary of evidence	19	Summarize the main results (including an overview of concepts, themes, and types of evidence available), link to the review questions and	13-14

SECTION	ITEM	PRISMA-ScR CHECKLIST ITEM	REPORTED ON PAGE #
		objectives, and consider the relevance to key groups.	
Limitations	20	Discuss the limitations of the scoping review process.	14-15
Conclusions	21	Provide a general interpretation of the results with respect to the review questions and objectives, as well as potential implications and/or next steps.	15
**FUNDING**			
Funding	22	Describe sources of funding for the included sources of evidence, as well as sources of funding for the scoping review. Describe the role of the funders of the scoping review.	17

JBI = Joanna Briggs Institute; PRISMA-ScR = Preferred Reporting Items for Systematic reviews and Meta-Analyses extension for Scoping Reviews.
* Where *sources of evidence* (see second footnote) are compiled from, such as bibliographic databases, social media platforms, and Web sites.
† A more inclusive/heterogeneous term used to account for the different types of evidence or data sources (e.g., quantitative and/or qualitative research, expert opinion, and policy documents) that may be eligible in a scoping review as opposed to only studies. This is not to be confused with *information sources* (see first footnote).
‡ The frameworks by Arksey and O'Malley (6) and Levac and colleagues (7) and the JBI guidance (4, 5) refer to the process of data extraction in a scoping review as data charting.
§ The process of systematically examining research evidence to assess its validity, results, and relevance before using it to inform a decision. This term is used for items 12 and 19 instead of "risk of bias" (which is more applicable to systematic reviews of interventions) to include and acknowledge the various sources of evidence that may be used in a scoping review (e.g., quantitative and/or qualitative research, expert opinion, and policy document).

From: Tricco AC, Lillie E, Zarin W, O'Brien KK, Colquhoun H, Levac D, et al. PRISMA Extension for Scoping Reviews (PRISMAScR): Checklist and Explanation. Ann Intern Med. 2018;169:467–473. doi: 10.7326/M18-0850.
